# DNA Transposons: Nature and Applications in Genomics

**DOI:** 10.2174/138920210790886871

**Published:** 2010-04

**Authors:** Martín Muñoz-López, José L. García-Pérez

**Affiliations:** Andalusian Stem Cell Bank, Center for Biomedical Research, University of Granada, Avda. del Conocimiento s/n, Armilla, 18100, Granada, Spain

**Keywords:** Transposable elements, DNA transposons, Tc1/mariner elements, Sleeping Beauty, piggyBac, Tol2, insertional mutagenesis, transgenesis.

## Abstract

Repeated DNA makes up a large fraction of a typical mammalian genome, and some repetitive elements are able to move within the genome (transposons and retrotransposons). DNA transposons move from one genomic location to another by a cut-and-paste mechanism. They are powerful forces of genetic change and have played a significant role in the evolution of many genomes. As genetic tools, DNA transposons can be used to introduce a piece of foreign DNA into a genome. Indeed, they have been used for transgenesis and insertional mutagenesis in different organisms, since these elements are not generally dependent on host factors to mediate their mobility. Thus, DNA transposons are useful tools to analyze the regulatory genome, study embryonic development, identify genes and pathways implicated in disease or pathogenesis of pathogens, and even contribute to gene therapy. In this review, we will describe the nature of these elements and discuss recent advances in this field of research, as well as our evolving knowledge of the DNA transposons most widely used in these studies.

## INTRODUCTION

Eukaryotic genomes contain an abundance of repeated DNA, and some repeated sequences are mobile. Transposable elements (TEs) are defined as DNA sequences that are able to move from one location to another in the genome. TEs have been identified in all organisms, prokaryotic and eukaryotic, and can occupy a high proportion of a species’ genome. For example, transposable elements comprise approximately 10% of several fish species, 12 % of the *C. elegans* genome [1, 2], 37% of the mouse genome [3], 45% of the human genome [4], and up to >80% of the genome of some plants like maize [5]. From bacteria to humans, transposable elements have accumulated over time and continue to shape genomes through their mobilization.

The mobilization of TEs is termed transposition or retrotransposition, depending on the nature of the intermediate used for mobilization. There are several ways in which the activity of TEs can positively and negatively impact a genome; for example, TE mobilization can promote gene inactivation, modulate gene expression or induce illegitimate recombination. Thus, TEs have played a significant role in genome evolution. However, from a strictly theoretical point of view, TEs can be considered as *selfish* DNA or *junk* DNA, and the existence of these elements in a genome represents the fight between selfish DNA (to be perpetuated) and the host (to curtail their spread and its consequences).

As TEs make up a large percentage of genome volume, it is hypothesized that they have participated in changes of genome size during speciation and evolution, as reported in plants [6], *Drosophila* or primates [7-9]. The trigger(s) for TE-induced genome size increases is not clearly known, although it is thought that stress could be implicated in the amplification of TEs [10]. TEs are able to produce various genetic alterations upon insertion as a consequence of the transposition process (insertions, excisions, duplications or translocations in the site of integration). For example, DNA transposons can inactivate or alter the expression of genes by insertion within introns, exons or regulatory regions [11-15]. In addition, TEs can participate in the reorganization of a genome by the mobilization of non-transposon DNA [16-18] or by acting as recombination substrates. This recombination would occur by homology between two sequences of a transposon located in the same or different chromosomes, which could be the origin for several types of chromosome alterations [19]. Indeed, TEs can participate in the loss of genomic DNA by internal deletions [20] or other mechanisms [21, 22].

The reduction in fitness suffered by the host due to transposition ultimately affects the transposon, since host survival is critical to perpetuation of the transposon. Therefore, strategies have been developed by host and transposable elements to minimize the deleterious impact of transposition, and to reach equilibrium. For example, some transposons tend to insert in nonessential regions in the genome, such as heterochromatic regions [23-26], where insertions will likely have a minimal deleterious impact. In addition, they might be active in the germ line or embryonic stage [27-29], where most deleterious mutations can be selected against during fecundation or development, allowing only non-deleterious or mildly deleterious insertions to pass to successive generations. New insertions may also occur within an existing genomic insertion to generate an inactive transposon, or can undergo self-regulation by *overproduction-inhibition* (see below). On the other hand, host organisms have developed different mechanisms of defense against high rates of transposon activity, including DNA-methylation to reduce TE expression [30-33], several RNA interference mediated mechanisms [34] mainly in the germ line [35, 36], or through the inactivation of transposon activity by the action of specific proteins [37-39].

In some cases, transposable elements have been “domesticated” by the host to perform a specific function in the cell [40]. A well-known example are RAG proteins, which participate in V(D)J recombination during antibody class switching, and exhibit a high similarity to DNA transposons, from which these proteins appear be derived [41-45]. Another example is the centromeric protein CENP-B, which seems to have originated from the *pogo*-like transposon [46]. The analogous human *mariner* *Himar1* element has been incorporated into the SETMAR gene, which consists of the histone H3 methylase gene and the *Himar1* transposase domain. This gene is involved in the non-homologous end joining pathway of DNA repair, and has been shown to confer resistance to ionizing radiation [47]. From a genome wide view, it has been estimated that ~25% of human promoter regions and ~4% of human exons contain sequences derived from TEs [48, 49]. Thus, we are likely underestimating the rate of domestication events in mammalian genomes.

**Fig. (1):**
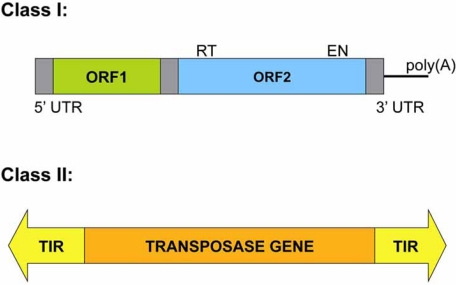
*Classes of Transposable Elements (TEs).* A Class I element (clade LINE-1) consist of a 5’-UTR with internal promoter activity, and two Open Reading Frames (ORFs). ORF1 encodes a *nucleic acid binding protein*, and ORF2 encodes a protein with Endonuclease (EN) and Reverse Transcriptase (RT) activity, lacks Long Terminal Repeats (LTR), and ends in a poly(A) tail (reviewed in [51]). Class II elements consist of a transposase gene flanked by Terminal Inverted Repeats (TIRs).

A type of TE, RNA transposons (Class I), function *via* reverse transcription of an RNA intermediate (replicative mechanism) and can be further subdivided in two main groups depending on the presence of *Long Terminal Repeats* (LTR) flanking the retroelement main body (Fig. **1**). LTR retrotransposons are similar in structure and life cycle to retroviruses, and their biology has been recently reviewed [50]. Additionally, the biology and impact of non-LTR retrotransposons in mammalian genomes has been reviewed extensively (see [51], for a recent review) as well as their potential use as mutagens in genomics [52]. Thus, no Class I TEs will be reviewed in this manuscript, although they posses some unique characteristics that may be very useful in genomics studies.

DNA transposons (Class II) generally move by a cut-and-paste mechanism in which the transposon is excised from one location and reintegrated elsewhere. Most DNA transposons move through a non-replicative mechanism, although there are exceptions (see below). DNA transposons consist of a transposase gene that is flanked by two *Terminal Inverted Repeats* (TIRs) (Fig. **1**). The transposase recognizes these TIRs to perform the excision of the transposon DNA body, which is inserted into a new genomic location (see below for further details). Upon insertion, target site DNA is duplicated, resulting in *Target Site Duplications* (TSDs), which represent a unique hallmark for each DNA transposon. DNA transposons are classified into different families depending on their sequence, TIRs, and/or TSDs. The families in Subclass I are: Tc1/*mariner*, PIF/*Harbinger*, hAT, Mutator, Merlin, Transib, P, *piggyBac* and CACTA. *Helitron* and *Maverick* transposons belong to a different subclass (Subclass II), since they are replicated and do not perform double-strand DNA breaks during their insertion (see below).

Within both classes of TEs (Class I and II) we can find non-autonomous elements (i.e., do not encode proteins required for their mobilization), which are presumably dependent on autonomous transposons for mobility. As an example,* Miniature Inverted-repeat Transposable Elements* (MITEs) are short (80-500 bp) DNA transposon-like elements present in large numbers in many eukaryotes, particularly plant species [53, 54], and occasionally in bacteria [55, 56]. Although they have TIRs and are flanked by TSDs, lack transposase coding potential and are thus presumably dependent on autonomous DNA transposons for their mobilization.

In the following sections, we will describe and review several DNA transposon families, from their nature to their applications as genomic tools.

### Superfamily Tc1/*mariner*

The elements from the superfamily Tc1/*mariner* are probably the most widely distributed family of TEs in nature, represented in such diverse taxa as rotifers, fungi, plants, fish and mammals [57-59]. Despite this fact, the vast majority harbor multiple inactivating mutations and only ten naturally occurring elements are known to be active: Tc1 and Tc3 from *C. elegans* [60, 61], *Minos* from *Drosophila hydei* [62], *Mos1* from *D. mauritiana* [63, 64], *Famar1* from European Earwig (*Forficula auricularia*) [65], *Osmar5* from rice (*Oryza sativa*) [66], *Fot1* and *Impala* from the fungus *Fusarium oxysporum* [67, 68], *ISY100* isolated in bacteria [69], and *Mboumar-9* from the ant *Messor bouvieri* [70]. In addition, four well-characterized active Tc1/*mariner* transposons have been reconstructed from inactive elements: *Sleeping Beauty* from salmonid-type fishes [71], *Himar1* from the Horn Fly (*Hematobia irritans*) [72], *Frog Prince* from the frog *Rana pipiens* [73] and *Hsmar1*, incorporated into the SETMAR gene,** from *H. sapiens* [74-76].

**Fig. (2):**
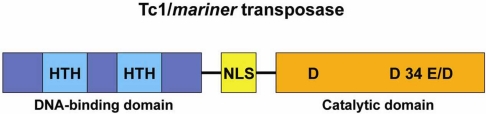
*Structure of Tc1/mariner transposase.* Schematic representation of the Tc1/*mariner* transposase, which contains a DNA-binding domain with the *Helix-Turn-Helix* motif (HTH), a *Nuclear Localization Signal* (NLS) and a catalytic domain with the DDE or DDD motif.

Tc1/*mariner* elements are between 1 and 5 kb in length, and encode a transposase of 282 to 345 amino acids which is flanked by two TIRs that can vary between 17 to 1100 bp in length [58, 77]. The transposase proteins from different Tc1/*mariner* elements are not very similar in sequence, but all of them harbor two characteristic domains: an amino-terminal region containing the helix-turn-helix (HTH) motif necessary for recognition and binding of TIRs, and a carboxy-terminal domain harboring the catalytic motif constituted by three amino acids, DDD in the case of *mariner*-like elements, or DDE in the case of Tc1-like elements (Fig. **2**). The first and second aspartate residues are separated by 92 amino acids, whereas the distance between the second and third residue is variable, between 31 and 39 amino acids in the different families from the superfamily Tc1/*mariner* [78]. Other motifs harbored by the transposase are the *Nuclear Localization Signal* (NLS), indispensable for transposase transport through the nuclear membrane [78], and the WVPHEL linker motif, which might participate in the interaction between transposase monomers [78].

Tc1/*mariner* elements can be further sub-classified in seven different groups or families: *ma*T elements (DD37D), isolated from *Bombyx mori*, *C. elegans* and *C.* *briggsae* [79]; DD37E elements isolated from mosquito and rotifers [59, 80]; Tc1-like elements (DD34E) isolated from insects, nematodes and vertebrates [71, 81-84]; *Gambol* elements (DD34E), phylogenetically distinct from the group above and isolated from mosquito [85]; DD39D elements isolated only in plants [86]; and *mariner*-like elements (DD34D). The *mariner* family is probably the most widely distributed family of transposons in nature, represented in such diverse taxa as fungi, ciliates, rotifers, insects, nematodes, plants, fish and mammals [57-59]. The phylogenetic analysis of theses elements define at least five clear sub-families: *mauritiana*, *cecropia*, *mellifer*/*capitata*, *irritans* and *elegans*/*briggsae*.

### Transposition Mechanism

**Fig. (3):**
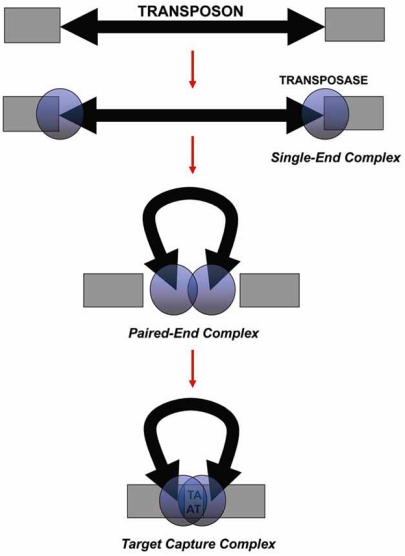
*Transposition steps.* Representation of the transposition mechanism performed by the transposase proposed for Tc1/*mariner* elements. The process begins with the binding of two transposase monomers to the TIRs, forming the *Single-End Complex*. Then, the transposon ends are brought together by both transposase monomers that form a dimer, generating the *Paired-End Complex*, and transposon excision takes places. Finally, the transposase dimer recognises a TA dinucleotide, joins it, and forms the *Target Capture Complex* to carry out the insertion.

**Fig. (4):**
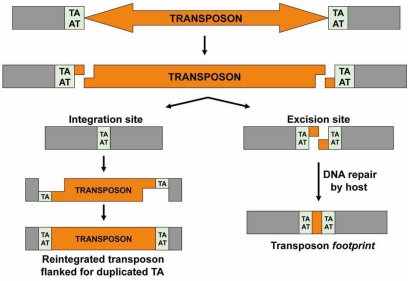
*Cut and paste reaction.* Representation of cut-and-paste reaction in which the transposon is excised from one site and reintegrated at a TA target dinucleotide. Upon insertion, the TA dinucleotide is duplicated generating the *Target Site Duplication* (TSD). Then, the host will repair the excision site. If this repair is carried out by *nonhomologous end-joining* (NHEJ), a transposon *footprint* is generated.

The mobilization of Tc1/*mariner* elements is a non-replicative transposition process that operates by a cut-and-paste mechanism (Figs. **3** and **4**) and consists of the following steps:

1. Two transposase molecules recognize the TIRs and bind to them via their HTH motifs, forming the Single-End Complex (SEC) (Fig. **3**).
2. Both transposases cleave the 5’-ends of the TIRs by hydrolysis of the phosphodiester bond to liberate the non-transferred strands (5’-P extremes), which do not participate further in the transposition process (Fig. **4**).
3. The two transposase molecules interact and bring together the transposon ends to form the *Paired-End Complex* (PEC) generating a transposase dimer (Fig. **3**). At this point, the phosphodiester bond undergoes a hydrolysis in the 3’-ends to produce the transferred strands (3’-OH extremes) (Fig. **4**).
4. The PEC binds to target DNA forming the *Target Capture Complex*, at which insertion takes place (Fig. **3**). The target in Tc1/*mariner* elements is any TA dinucleotide. Therefore, the transposase selects a random TA where the transposon insertion will be carried out. The 5’-end in the target DNA undergoes a nucleophilic attack from the transposon *transferred strands* 3’-OH. The gaps in the transposon 5’-ends are filled by the host, generating canonical TSDs flanking the new transposon insertion (Fig. **4**).

None of the transposition steps described above require energy (in the form of the cofactor ATP), since the necessary energy to form the phosphodiester bonds in the integration process comes from the cleavage reaction of target DNA (exergonic reaction) [87-89]. Indeed, the catalytic motif DDE/D in the transposase carries out both excision and insertion reactions during transposition. However, the DDE/D motif needs to interact with a divalent cation to perform the transposition reaction. Although the physiological ion is Mg^2+^, the transposase can also use the cofactor Mn^2+^, which seems to cause a relaxation in target site specificity. This has been seen for many transposition systems and is supported by experimental evidence that indicates that Mn^2+^permits more flexible DNA strand positioning in the active site than does Mg^2+^ [74, 90].

Consistent with these data, transposition of Tc1/*mariner* elements requires no proteins or cofactors other than Mg^2+^and the transposase itself. Indeed, elements from this superfamily are able to perform transposition *in vitro*, when provided the right pH and salt conditions, a donor and target DNA, Mg^2+^ or Mn^2+^, and an active transposase protein [70, 72, 91]. Therefore, this fact confirms that Tc1/*mariner* elements are not dependent on host factors to mediate their mobility, making them excellent tools for genomic manipulation in non-native hosts (see below). However, in some circumstances, it has been reported that the transposition efficiency can be affected by the cellular environment [92].

To complete a round of transposition, the DNA double strand breaks (DSBs) left behind by the Tc1/*mariner* transposons upon excision must be repaired by the host cellular machinery. One possible pathway of DSB repair is *homologous recombination* (HR), either using the homologous chromosome (or the sister chromatid) or a homologous sequence on the same chromosome as a template. In the first case, the result is the regeneration of a new copy of the transposon [93]. In the second case, repair occurs by single-strand annealing, generating a deletion in the DNA flanking the excision site [93]. Another possibility is to repair the DSBs through the *Non-Homologous End-Joining* (NHEJ) DNA repair pathway, which leads to the generation of a transposon *footprint* flanked by the TA duplication [94, 95]. The choice of DSB repair is likely dictated by the host, as different organisms are more prone to repair DSBs lesions through either HR or NHEJ.

### Regulation and Control of Transposition

Transposition is potentially deleterious to the host as well as the transposon, whose replication and propagation depend on the survival of their host. Thus, the development of ways to decrease the impact of transposition on host fitness is beneficial for both host and transposon. Some of the known strategies for transposon control are the following:

#### Overproduction Inhibition (OPI)

The transposase itself can act as a transposition inhibitor, as when it exceeds a threshold concentration, transposon activity is decreased. This fact has been observed in *Tc1/mariner* elements [96, 97], although the nature of this mechanism is not clear. It has been suggested that transposase monomers could form inactive or less active oligomers, thus decreasing the activity of the transposition process [96, 97]. When the copy number of these elements increases in the host genome, the production of transposase is also increased, and through OPI the mobilization of the transposon is reduced.

#### Vertical Inactivation

Although Tc1/*mariner* elements are widespread in nature, the vast majority harbor multiple inactivating mutations and only a few naturally occurring elements are known to be active (see above). It has been suggested that this is the result of selective pressure to reduce damage to the host genome [98]. In addition, inactive elements could produce inactive transposases that would impede the transposition of active elements, by OPI or by competition with the active transposases for TIRs. As two functional transposase molecules are necessary to perform transposition, inactive transposase proteins act as dominant negative inhibitors of transposition [96, 99]. On the other hand, inactive elements with active TIRs can recruit active transposase to mediate their mobilization. This phenomenon could explain the replacement of active elements by inactive elements, which seems to have occurred in many species during the course of evolution [53].

#### Other Mechanisms

As mentioned above, the host can develop different mechanisms to decrease the activity of transposons. One way used by the host to silence a Tc1/*mariner* element is DNA methylation, thereby preventing its transcription [100], or using post-transcriptional silencing mechanisms such as RNA interference [101, 102].

### Life Cycle and Horizontal Transfer

**Fig. (5):**
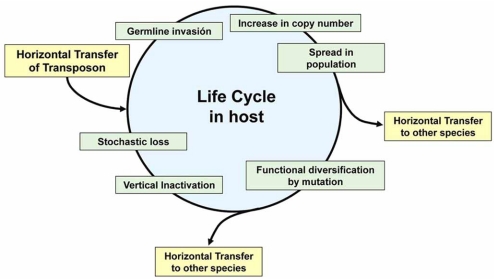
*Life cycle of* Tc1/*mariner.* Shown is the evolutionary life cycle proposed for Tc1/*mariner* elements. The figure has been adapted from Miskey *et al.*, [92].

TEs are parasitic DNAs whose only function is to replicate and propagate themselves. When a transposon invades a new host, it must colonize the germline genome to persist in the population. Then, it will increase in copy number [103], and persists in the genome until, by *vertical inactivation*, all transposon copies become inactive and remain only as fossils. These inactive elements may even disappear by genetic drift [98]. To escape this cycle, a transposon must invade a new species, or extends to multiple species. In other words, to ensure its survival, the transposon must pass to a new genome by *Horizontal Transfer*, and begin its life cycle again (Fig. **5**).

As discussed previously, Tc1/*mariner* elements do not require specific factors from the host to perform the transposition process, and therefore are not restricted to one specific host. Indeed, many cases of horizontal transfer between different hosts have been proposed for these elements. Examples include transfer between marine crustaceans [104], between insects from different orders [105, 106], and even between organisms from different phyla, as divergent as human and a parasitic nematode [107]. However, it is not known how these elements are able to invade new genomes. Potential vectors that might be implicated in this horizontal transfer are external parasites, such as mites, which seems to be the vehicle for the horizontal transfer of P elements in *Drosophila* [108], or internal parasites such as viruses [103].

### Tc1/*mariner* Transposons as Genetics Tools

*Sleeping Beauty* (SB) is the Tc1/*mariner* element most widely used as a genetic tool. It is a synthetic transposable element reconstructed from defective copies of eight salmon species by reverse engineering [83]. SB is active in species ranging from protozoa to vertebrates, including frogs, fish, mice, rats or humans [109]. The hyperactive version of SB, *SB100X,* exhibits approximately a 100-fold increase in efficiency when compared to the first generation of SB transposase, facilitating robust stable gene transfer in vertebrates [110]. Therefore, SB represents a promising system for gene transfer in vertebrates (somatic and germ line), embryonic stem cells, and many other cultured cell lines [110, 111].

**Fig. (6):**
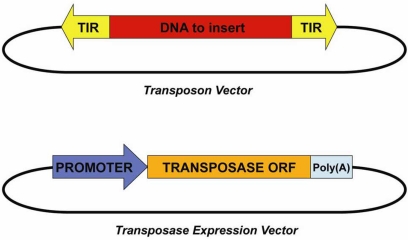
*DNA-Transposon System.* The *Transposon Vector*, consisting of the DNA of interest flanked by transposon TIRs, and the *Transposase Expression Vector*, harbouring the transposase gene placed downstream of a strong promoter.

The SB transposon system, similar to other DNA transposons, consists of two components (Fig. **6**): the *SB transposon vector*, which contains the gene to be mobilized flanked by SB TIRs, and the *SB transposase expression vector,* which is the transposase mRNA or an expression plasmid. The *SB transposase expression vector* contains the SB transposase *open reading frame* (ORF) between a strong promoter (ubiquitous or cell-type restricted) and a poly(A) signal. To achieve transposition of SB, the two components of the system are introduced in the host (transfection in cell cultures, injection into fertilized eggs, injection in live animals, etc.) where insertion takes places. The SB system has been tested in several fish species, the frog *Xenopus*, rat, mouse and in cultured human cell lines [110, 112-114].

In humans, the SB transposon system was initially used in human T cells, resulting in stable gene transfer and expression of the reporter gene [111]. The novel hyperactive *SB100X* has been tested in primary human CD34-positive hematopoietic stem cells, resulting in stable gene expression [110]. Furthermore, transgenic mice have been generated by co-injecting the *SB transposon vector* with the SB transposase mRNA into fertilized oocytes, some of which gave rise to transgenic offspring [110]. Additionally, SB has also been used in functional genetic screens in mammals for the identification of genes implicated in diseases such as cancer. SB is used to induce insertional mutagenesis, and candidate genes identified through the analysis of insertion sites in tumors vs control tissues (in gain of function studies [115, 116], reviewed in [117]).

Although *Sleeping Beauty* is currently the most promising gene transfer system for vertebrate cells within the Tc1/*mariner* superfamily, other transposons from this family have been used as genomic tools as well. *Frog Prince* was reconstructed from the Northern Leopard frog, *Rana pipiens*, and is characterized by the presence of 214 bp-long TIRs flanking the transposase gene (which harbors a DD34E catalytic domain, see above). *Frog Prince* shows preference for intronic insertions, and is very efficient in gene trapping experiments conducted in tissue culture cells [73]. Furthermore, *Frog Prince* has been tested in zebrafish embryos and other cultured vertebrate cell lines [73]. Similarly, the transposon *Minos*, isolated from *Drosophila hydei*, is 1.8 kb in length, has 254 bp-long TIRs and a two-exon transposase gene (60 bp-long intron) with the catalytic domain DD34D. This transposon has preference for genes, inserting mostly into introns, and has been tested in cultured human cells [118], mouse tissues [119] and the sea squirt *Ciona intestinalis* [120]. Another example is *Himar1* (also with a DD34D transposase), reconstructed from *Haematobia irritans*, which has been used in screens to identify genes implicated in bacterial pathogenicity by insertional mutagenesis [121-123], and in cultured human cells [124]. In addition, there are other Tc1/*mariner* transposons that are active, but have not been tested in cells yet; for example, *Mboumar-9*,** a new naturally active *mariner* transposon from ant, which shows robust efficiency of transposition *in vitro* [70].

### Superfamily *piggyBac*

*piggyBac* is a DNA transposon identified in the genome of the Cabbage Looper moth (*Trichoplusia ni*). Much of its biology is shared with Tc1/*mariner* elements, including transposition mechanism, control, and life cycle. Related *piggyBac* transposable elements have been found in plants, fungi and animals, including humans [125], although they are probably inactive due to mutation. *piggyBac* is 2.4 kb in length, contains 13 bp TIRs, and additional 19 bp internal inverted repeats located asymmetrically with respect to the ends [126]. Its target insertion site is TTAA and it harbors a single ORF (1.8 kb) that encodes a functional transposase, although the DNA-binding domain and catalytic core have not yet been defined. The transposase from *piggyBac* has been optimized to generate a more active transposition system [127]. This transposon has been used in such diverse organisms as protozoa, planaria, insects and mammals, including human cells [128-132].

*piggyBac* represents a versatile gene-trap vector for transgenesis in insects, being the most widely used transposon system for germline transformation in these organisms (dipteran, hymenopteran, coleopteran and lepidopteran species). It is an important tool to generate modified insects carrying lethality or sterility genes by transgenesis for plague control and thus pest control [133-135]. In mammals, the *piggyBac* system has been used for different applications, such as germline or somatic mutagenesis and gene therapy. It has been used to mediate gene transfer in human cells [132] and recently to generate transgene-free induced pluripotent (iPS) stem cells from mouse cells [131].

### Superfamily hAT

DNA transposons from the superfamily hAT (*hobo*/*Ac*/*Tam3*) have been isolated in eukaryotes, are 2.5 to 5 kb in length, and encode a transposase harboring a catalytic DDE motif and a DNA binding domain BED zinc finger (named after *Drosophila* proteins DEAF and DREF) [136, 137]. In *hobo*/*Ac*/*Tam3* transposons, the** transposase gene is flanked by TIRs of 5 to 27 bp in length, and the TSDs of these elements consist of heterogenic sequences of 8 bp in length. A member from this family widely used as a genetic tool is *Tol2*, which was the first active autonomous transposon isolated in vertebrate species [138, 139]. This element was identified in Medaka fish (*Oryzias latipes*) where it had generated a mutation in the tyrosinase gene, resulting in albino mutant fish. *Tol2* is 4.7 kb in length and consists of two TIRs of variable length flanking the transposase gene which is made up of four exons [140]. It has also been engineered for improved efficiency to facilitate its use as a tool for enhancer trap screens in vertebrates to identify genes implicated in different functions and pathways [141-143]. *Tol2* can have a cargo capacity of more than 10 kb [144, 145], and its integration preference is not clear, although similarly to other hAT elements, it could have preference for 5’ regions of genes [146]. This system has been used in different vertebrates such as zebrafish and *Xenopus*, chicken embryos, and cultured vertebrate cells, including human stem cells [141, 147-149].

### Transposon System Characteristics

In the following section, we will discuss the most useful characteristics of each DNA transposon as well as their known limitations.

There are many ways to manipulate an organism’s genome (somatic or germline), and viral delivery systems applied in gene therapy have several disadvantages when compared to transposon vectors. For example, viral vectors may induce a destructive immune response [150, 151], their production is difficult and expensive [152, 153], they prefer to integrate within 5’UTR regions of genes which may induce oncogenesis [150, 154, 155], and they have a relatively limited cargo capacity (less than 8 kb in lentivirus, retrovirus or adeno-associated viral vectors) [150], among others. In contrast, transposon systems are inexpensive and easier to purify, and are non-inmunogenic [156-158]. In addition, they permit elimination of the transgene and, in some cases such as *piggyBac*, can be excised without leaving notable genetic alterations [131]. Unfortunately, relative to viral systems, DNA transposons are less efficient for gene transfer. However, the efficiencies of newly developed transposon systems such as *piggyBac* and *SB100X* are comparable to those of viruses [110, 127]. In addition, with a DNA transposon system as SB, almost 70% of the integrations occur in intergenic regions; they do not exhibit targeting of the 5’ region of genes as occurs with viruses [159, 160].

Among the characteristics that distinguish DNA transposon systems as biotechnical tools, we highlight:

### Capacity for Cargo

Transposon insertion efficiency can vary depending on the size of the gene to be transferred. Tc1/*mariner* elements are notably affected by this factor, since an increase in cargo size decreases the efficiency of transposition in cultured cells [161]. In contrast, *piggyBac* or *Tol2* transposons are more tolerant in their capacity for cargo. In *piggyBac,* when the cargo approaches 9 kb the efficiency decreases in pronucleus-injected mice [162], and in *Tol2* the efficiency begins to drop off only when the cargo is higher than 10 kb [144]. To overcome this limitation in the SB system, a “sandwich SB vector” has been designed, which consists of two complete SB transposons flanking the gene to be mobilized, increasing the number of SB binding sites and thereby improving the efficiency of transposition for transgenes longer than 10 kb [163].

### Integration Site Preference

Integration site preference is an important consideration when choosing a transposon system for a given application. For example, *piggyBac* has preference for transcription units, with insertions primarily targeting introns [132]. On the other hand, SB prefers heterochromatin over actively transcribed genes [26, 159], and when it does insert into genes, it prefers intronic sequences. Finally, superfamily hAT members like *Tol2* seem prone to insert within 5’ regions of genes [146]. The integration site preference is likely dictated by the transposase protein, and SB as well as other Tc1/*mariner* elements seem to have structural preferences with regards to their integration site [164, 165]. On the other hand, *piggyBac* inserts in its target TTAA without any other apparent requirements [166, 167]. Thus, depending on the study, both SB and *piggyBac* can be useful systems. In the case of mutagenesis screens, it is preferable for the transposon to insert into genes, whereas gene therapy protocols require a system with less affinity for insertion within genes and, in general, low-risk chromosomal regions. However, integration within heterochromatin (as observed for SB, [26]) has the disadvantage of typically producing low levels of transgene expression [168].

**Fig. (7):**
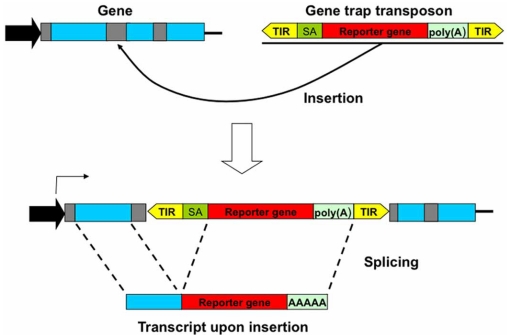
*Gene Trap Transposons.* A gene-trap designed to disrupt a gene, consisting of the transposon TIRs flanking a *strong splice* acceptor (SA) site followed by a reporter gene and a strong poly(A) signal. Therefore, if this transposon inserts into an intron of a gene (introns in grey; exons in blue), the inserted reporter will provoke a *mis*-splicing process and as a result the trapped gene is inactivated.

In functional genomic studies, it is often desirable to inactive genes by insertional mutagenesis by transposons. If the transposon insertion takes place within an intron, splicing would likely render such an insertion irrelevant. To avoid this situation, a *splice acceptor* followed by the reporter gene and a poly(A) tail may be included in the transposon. In this way, splicing is altered, leading to the fusion of the trapped gene and reporter gene downstream (Fig. **7**). Thus, the trapped gene remains inactivated and the reporter gene is expressed. In sum, for insertional mutagenesis studies, both *Tol2* and *piggyBac* are superior to SB, while for gene therapy SB is theoretically more secure than either *Tol2* or *piggyBac* transposon systems.

### Local Hopping

Like others Tc1/*mariner* elements, SB tends to insert in the vicinity of the donor locus. This phenomenon is known as *Local Hopping* and seems to be a property of the Tc1/*mariner* family, as well as other DNA transposons including P elements or *Ac* elements. For example, SB shows a much larger local transposition interval (5-15 Mb) than P elements (100 kb). Differences in the range of *Local Hopping* have been observed for the same transposon, depending on host species and chromosomal location of the donor site [169-172]. In some cases, this phenomenon could be exploited to produce insertions in a limited chromosomal region. In the opposite case, when it is necessary to extend the mutagenesis region, a solution could be to establish several donor loci. In contrast, recent reports have indicated less *Local Hopping* for *piggyBac* [162], although further studies are required to truly determine its *Local Hopping* constrains.

### Overproduction Inhibition

The Overproduction Inhibition (OPI) phenomenon, as described previously, consists of decreasing transposition due to high transposase concentration. This phenomenon appears in Tc1/*mariner* elements and is variable depending on the transposon from this family [96, 163], whereas in *piggyBac* and *Tol2* the OPI has not yet been described [132, 141]. In fact, this is the main limitation of the SB system. Using *piggyBac,* it is also possible to use a transposase-transposon vector, which results in a 2-fold higher activity in human cells relative to protocols in which the transposon and transposase plasmids are transfected separately. Therefore, OPI represents a disadvantage for gene transfer in Tc1/*mariner* elements, but not for transposons from others families such as *piggyBac* and hAT. However, the mechanism responsible for OPI is not clearly understood.

### The Perfect Transposon System for Genomics

DNA transposon systems represent an important alternative to viral systems for gene therapy studies, and they have several advantageous properties that make them very promising tools for a wide variety of genomic studies (Table **1**).

If we compare the characteristics of the most frequently used DNA transposon systems, SB and *piggyBac*, we believe that *piggyBac* has some advantages over SB, such as its high efficiency of insertion, the lack of OPI, non-*local hopping,* and a relatively high tolerance for cargo size (9-14 kb) [162] (Table **1**). In contrast, SB undergoes OPI, *local hopping* and its efficiency of insertion decreases as a function of transgene length. However, the new hyperactive SB version, *SB100X*, seems to have a higher efficiency of insertion than *piggyBac* [110], unlike previous SB versions [127]. Another advantage of *piggyBac* is that it does not leave “*footprint*” upon excision, unlike DNA transposons such as Tc1/*mariner* elements. The “*footprint*” of SB is TAG(T/A)CTA, whereas the *piggyBac* target site is repaired to the original sequence [162], which allows removal of the inserted transposon leaving the genome without any sequence alteration, a very important characteristic for applications in gene therapy. For example, *piggyBac* has been used to generate iPS cells, and later the reprogramming factors have been removed from the genome of iPS cells by re-expressing the transposase [131].

Taking into consideration the virtues and disadvantages of current DNA transposons for genomics studies, the hypothetical “perfect” transposon system would be: a high-efficiency system comparable with that of viral vectors or higher, that does not manifest OPI, that lacks local hopping (although in some cases this could be useful), with a high capacity for cargo, that leaves no-footprint upon insertion, and that induces the lowest possible level of mutations and chromosomal rearrangements. Among some other characteristics to consider, the preference of insertion site could be variable depending on the goal of the study. If the purpose is insertional mutagenesis for a screen of gene function, it would be necessary that the transposon has a preference for insertion into genes, as do *piggyBac* and likely* Tol2*. However, in gene therapy protocols it is essential that the insertion occurs outside genes, as with SB, to avoid deleterious mutations or chromosomal alterations that could originate during integration–excision events.

At present, the transposon system that encompasses more of these characteristics is *piggyBac*, follow by SB. Although *Tol2* is similar to *piggyBac* in most aspects, the mobilization of *piggyBac* seems to be more efficient [173]. SB and *piggyBac* have been tested successfully in mammalian genomes, including humans, to carry out transgenesis and functional genomics studies. Therefore, by virtue of their natural characteristics acquired over the course of their evolution as genetic parasites or *selfish* DNA, DNA transposons constitute a promising tool to perform important advances in functional genomics studies, gene therapy approaches, and for the generation of animal models with Knock-Out in each gene contained in its genome. Many of the useful characteristics of DNA transposons have been improved, and efforts have been made to overcome their inherent disadvantages. Further research, however, is required to obtain *a perfect transposon system*. Despite potential limitations inherent to their “free life” in host genomes, among them the propensity to generate mutations or chromosomal rearrangements, we should emphasize that these characteristics have been an important catalyst for genomic variability, which ultimately represents the raw material of evolution. Although repeated DNA and TEs are sometimes considered *junk* DNA, they have and will continue to prove useful in many biotechnical applications, and will remain a motor for the evolution of species.

## Figures and Tables

**Table 1:** Characteristics of DNA Tranposons Used in Genomics

Transposon	Origin	Target	Integration Site Preference	Capacity for Cargo	Overproduction Inhibition	Local Hopping
*Sleeping Beauty* (Superfamily Tc1/*mariner*)	Salmon species (reconstructed)	TA	Intergenic regions	>10 Kb, efficiency decrease with size	Yes	High
*piggyBac* (Superfamily piggyBac)	*Trichoplusia ni*	TTAA	Transcription units (introns)	>9 Kb	Not observed	Low
*Tol2* (Superfamily hAT)	*Oryzias latipes* (Medaka fish)	Heterogenic sequence of 8 bp	Probably 5’ regions of genes	>10 Kb	Not observed	Low

## ABBREVIATIONS

DSB: = Double Strand Break
HR: = Homologous Recombination
HTH: = Helix-Turn-Helix
LTR: = Long Terminal Repeats
MITE: = Miniature Inverted-repeat Transposable Element
NHEJ: = Non Homologous End Joining
NLS: = Nuclear Localization Signal
OPI: = OverProduction Inhibition
ORF: = Open Reading Frame
PEC: = Paired-End Complex
SB: = Sleeping Beauty
SEC: = Single-End Complex
TE: = Transposable Element
TIR: = Terminal Inverted Repeats
TSD: = Target Site Duplication

## References

[1] Consortium C. e. S. (1998). Genome sequence of the nematode C. elegans: a platform for investigating biology. Science.

[2] Stein L D, Bao Z, Blasiar D, Blumenthal T, Brent M R, Chen N, Chinwalla A, Clarke L, Clee C, Coghlan A, Coulson A, D'Eustachio P, Fitch D H, Fulton L A, Fulton R E, Griffiths-Jones S, Harris T W, Hillier L W, Kamath R, Kuwabara P E, Mardis E R, Marra M A, Miner T L, Minx P, Mullikin J C, Plumb R W, Rogers J, Schein J E, Sohrmann M, Spieth J, Stajich J E, Wei C, Willey D, Wilson R K, Durbin R, Waterston R H (2003). The genome sequence of Caenorhabditis briggsae: a platform for comparative genomics. PLoS. Biol.

[3] Waterston R H, Lindblad-Toh K, Birney E, Rogers J, Abril J F, Agarwal P, Agarwala R, Ainscough R, Alexandersson M, An P, Antonarakis S E, Attwood J, Baertsch R, Bailey J, Barlow K, Beck S, Berry E, Birren B, Bloom T, Bork P, Botcherby M, Bray N, Brent M R, Brown D G, Brown S D, Bult C, Burton J, Butler J, Campbell R D, Carninci P, Cawley S, Chiaromonte F, Chinwalla A T, Church D M, Clamp M, Clee C, Collins F S, Cook L L, Copley R R, Coulson A, Couronne O, Cuff J, Curwen V, Cutts T, Daly M, David R, Davies J, Delehaunty K D, Deri J, Dermitzakis E T, Dewey C, Dickens N J, Diekhans M, Dodge S, Dubchak I, Dunn D M, Eddy S R, Elnitski L, Emes R D, Eswara P, Eyras E, Felsenfeld A, Fewell G A, Flicek P, Foley K, Frankel W N, Fulton L A, Fulton R S, Furey T S, Gage D, Gibbs R A, Glusman G, Gnerre S, Goldman N, Goodstadt L, Grafham D, Graves T A, Green E D, Gregory S, Guigo R, Guyer M, Hardison R C, Haussler D, Hayashizaki Y, Hillier L W, Hinrichs A, Hlavina W, Holzer T, Hsu F, Hua A, Hubbard T, Hunt A, Jackson I, Jaffe D B, Johnson L S, Jones M, Jones T A, Joy A, Kamal M, Karlsson E K, Karolchik D, Kasprzyk A, Kawai J, Keibler E, Kells C, Kent W J, Kirby A, Kolbe D L, Korf I, Kucherlapati R S, Kulbokas E J, Kulp D, Landers T, Leger J P, Leonard S, Letunic I, Levine R, Li J, Li M, Lloyd C, Lucas S, Ma B, Maglott D R, Mardis E R, Matthews L, Mauceli E, Mayer J H, McCarthy M, McCombie W R, McLaren S, McLay K, McPherson J D, Meldrim J, Meredith B, Mesirov J P, Miller W, Miner T L, Mongin E, Montgomery K T, Morgan M, Mott R, Mullikin J C, Muzny D M, Nash W E, Nelson J O, Nhan M N, Nicol R, Ning Z, Nusbaum C, O'Connor M J, Okazaki Y, Oliver K, Overton-Larty E, Pachter L, Parra G, Pepin K H, Peterson J, Pevzner P, Plumb R, Pohl C S, Poliakov A, Ponce T C, Ponting C P, Potter S, Quail M, Reymond A, Roe B A, Roskin K M, Rubin E M, Rust A G, Santos R, Sapojnikov V, Schultz B, Schultz J, Schwartz M S, Schwartz S, Scott C, Seaman S, Searle S, Sharpe T, Sheridan A, Shownkeen R, Sims S, Singer J B, Slater G, Smit A, Smith D R, Spencer B, Stabenau A, Stange-Thomann N, Sugnet C, Suyama M, Tesler G, Thompson J, Torrents D, Trevaskis E, Tromp J, Ucla C, Ureta-Vidal A, Vinson J P, Von Niederhausern A C, Wade C M, Wall M, Weber R J, Weiss R B, Wendl M C, West A P, Wetterstrand K, Wheeler R, Whelan S, Wierzbowski J, Willey D, Williams S, Wilson R K, Winter E, Worley K C, Wyman D, Yang S, Yang S P, Zdobnov E M, Zody M C, Lander E S (2002). Initial sequencing and comparative analysis of the mouse genome. Nature.

[4] Lander E S, Linton L M, Birren B, Nusbaum C, Zody M C, Baldwin J, Devon K, Dewar K, Doyle M, FitzHugh W, Funke R, Gage D, Harris K, Heaford A, Howland J, Kann L, Lehoczky J, LeVine R, McEwan P, McKernan K, Meldrim J, Mesirov J P, Miranda C, Morris W, Naylor J, Raymond C, Rosetti M, Santos R, Sheridan A, Sougnez C, Stange-Thomann N, Stojanovic N, Subramanian A, Wyman D, Rogers J, Sulston J, Ainscough R, Beck S, Bentley D, Burton J, Clee C, Carter N, Coulson A, Deadman R, Deloukas P, Dunham A, Dunham I, Durbin R, French L, Grafham D, Gregory S, Hubbard T, Humphray S, Hunt A, Jones M, Lloyd C, McMurray A, Matthews L, Mercer S, Milne S, Mullikin J C, Mungall A, Plumb R, Ross M, Shownkeen R, Sims S, Waterston R H, Wilson R K, Hillier L W, McPherson J D, Marra M A, Mardis E R, Fulton L A, Chinwalla A T, Pepin K H, Gish W R, Chissoe S L, Wendl M C, Delehaunty K D, Miner T L, Delehaunty A, Kramer J B, Cook L L, Fulton R S, Johnson D L, Minx P J, Clifton S W, Hawkins T, Branscomb E, Predki P, Richardson P, Wenning S, Slezak T, Doggett N, Cheng J F, Olsen A, Lucas S, Elkin C, Uberbacher E, Frazier M, Gibbs R A, Muzny D M, Scherer S E, Bouck J B, Sodergren E J, Worley K C, Rives C M, Gorrell J H, Metzker M L, Naylor S L, Kucherlapati R S, Nelson D L, Weinstock G M, Sakaki Y, Fujiyama A, Hattori M, Yada T, Toyoda A, Itoh T, Kawagoe C, Watanabe H, Totoki Y, Taylor T, Weissenbach J, Heilig R, Saurin W, Artiguenave F, Brottier P, Bruls T, Pelletier E, Robert C, Wincker P, Smith D R, Doucette-Stamm L, Rubenfield M, Weinstock K, Lee H M, Dubois J, Rosenthal A, Platzer M, Nyakatura G, Taudien S, Rump A, Yang H, Yu J, Wang J, Huang G, Gu J, Hood L, Rowen L, Madan A, Qin S, Davis R W, Federspiel N A, Abola A P, Proctor M J, Myers R M, Schmutz J, Dickson M, Grimwood J, Cox D R, Olson M V, Kaul R, Shimizu N, Kawasaki K, Minoshima S, Evans G A, Athanasiou M, Schultz R, Roe B A, Chen F, Pan H, Ramser J, Lehrach H, Reinhardt R, McCombie W R, de la Bastide M, Dedhia N, Blocker H, Hornischer K, Nordsiek G, Agarwala R, Aravind L, Bailey J A, Bateman A, Batzoglou S, Birney E, Bork P, Brown D G, Burge C B, Cerutti L, Chen H C, Church D, Clamp M, Copley R R, Doerks T, Eddy S R, Eichler E E, Furey T S, Galagan J, Gilbert J G, Harmon C, Hayashizaki Y, Haussler D, Hermjakob H, Hokamp K, Jang W, Johnson L S, Jones T A, Kasif S, Kaspryzk A, Kennedy S, Kent W J, Kitts P, Koonin E V, Korf I, Kulp D, Lancet D, Lowe T M, McLysaght A, Mikkelsen T, Moran J V, Mulder N, Pollara V J, Ponting C P, Schuler G, Schultz J, Slater G, Smit A F, Stupka E, Szustakowski J, Thierry-Mieg D, Thierry-Mieg J, Wagner L, Wallis J, Wheeler R, Williams A, Wolf Y I, Wolfe K H, Yang S P, Yeh R F, Collins F, Guyer M S, Peterson J, Felsenfeld A, Wetterstrand K A, Patrinos A, Morgan M J, de Jong P, Catanese J J, Osoegawa K, Shizuya H, Choi S, Chen Y J (2001). Initial sequencing and analysis of the human genome. Nature.

[5] SanMiguel P, Tikhonov A, Jin Y K, Motchoulskaia N, Zakharov D, Melake-Berhan A, Springer P S, Edwards K J, Lee M, Avramova Z, Bennetzen J L (1996). Nested retrotransposons in the intergenic regions of the maize genome. Science.

[6] SanMiguel P, Gaut B S, Tikhonov A, Nakajima Y, Bennetzen J L (1998). The paleontology of intergene retrotransposons of maize. Nat. Genet.

[7] Sheen F M, Levis R W (1994). Transposition of the LINE-like retrotransposon TART to Drosophila chromosome termini. Proc. Natl. Acad. Sci. USA.

[8] Frazer K A, Chen X, Hinds D A, Pant P V, Patil N, Cox D R (2003). Genomic DNA insertions and deletions occur frequently between humans and nonhuman primates. Genome Res.

[9] Locke D P, Segraves R, Carbone L, Archidiacono N, Albertson D G, Pinkel D, Eichler E E (2003). Large-scale variation among human and great ape genomes determined by array comparative genomic hybridization. Genome Res.

[10] Kalendar R, Tanskanen J, Immonen S, Nevo E, Schulman A H (2000). Genome evolution of wild barley(Hordeum spontaneum) by BARE-1 retrotransposon dynamics in response to sharp microclimatic divergence. Proc. Natl. Acad. Sci. USA.

[11] Jordan E, Saedler H, Starlinger P (1968). O0 and strong-polar mutations in the gal operon are insertions. Mol. Gen. Genet.

[12] Rubin G M, Kidwell M G, Bingham P M (1982). The molecular basis of P-M hybrid dysgenesis: the nature of induced mutations. Cell.

[13] Kazazian H H, Wong C, Youssoufian H, Scott A F, Phillips D G, Antonarakis S E (1988). Haemophilia A resulting from de novo insertion of L1 sequences represents a novel mechanism for mutation in man. Nature.

[14] Lerman D N, Feder M E (2005). Naturally occurring transposable elements disrupt hsp70 promoter function in Drosophila melanogaster. Mol. Biol. Evol.

[15] Clegg M T, Durbin M L (2003). Tracing floral adaptations from ecology to molecules. Nat. Rev. Genet.

[16] Moran J V, DeBerardinis R J, Kazazian H H (1999). Exon shuffling by L1 retrotransposition. Science.

[17] Sayah D M, Sokolskaja E, Berthoux L, Luban J (2004). Cyclophilin A retrotransposition into TRIM5 explains owl monkey resistance to HIV-1. Nature.

[18] Prak E T, Kazazian H H (2000). Mobile elements and the human genome. Nat. Rev. Genet.

[19] Kidwell M G, Lisch D R (2001). Perspective: transposable elements, parasitic DNA, and genome evolution. Evolution.

[20] Petrov D A, Hartl D L (1997). Trash DNA is what gets thrown away: high rate of DNA loss in Drosophila. Gene.

[21] Gilbert N, Lutz-Prigge S, Moran J V (2002). Genomic deletions created upon LINE-1 retrotransposition. Cell.

[22] Symer D E, Connelly C, Szak S T, Caputo E M, Cost G J, Parmigiani G, Boeke J D (2002). Human l1 retrotransposition is associated with genetic instability *in vivo*. Cell.

[23] Kidwell M G (1994). The Wilhelmine E. Key 1991 Invitational Lecture. The evolutionary history of the P family of transposable elements. J. Hered.

[24] Pimpinelli S, Berloco M, Fanti L, Dimitri P, Bonaccorsi S, Marchetti E, Caizzi R, Caggese C, Gatti M (1995). Transposable elements are stable structural components of Drosophila melanogaster heterochromatin. Proc. Natl. Acad. Sci. USA.

[25] Dimitri P, Arca B, Berghella L, Mei E (1997). High genetic instability of heterochromatin after transposition of the LINE-like I factor in Drosophila melanogaster. Proc. Natl. Acad. Sci. USA.

[26] Ikeda R, Kokubu C, Yusa K, Keng V W, Horie K, Takeda J (2007). Sleeping beauty transposase has an affinity for heterochromatin conformation. Mol. Cell. Biol.

[27] Levitt A, Emmons S W (1989). The Tc2 transposon in Caenorhabditis elegans. Proc. Natl. Acad. Sci. USA.

[28] Calvi B R, Gelbart W M (1994). The basis for germline specificity of the hobo transposable element in Drosophila melanogaster. EMBO J.

[29] Kano H, Godoy I, Courtney C, Vetter M R, Gerton G L, Ostertag E M, Kazazian H H (2009). L1 retrotransposition occurs mainly in embryogenesis and creates somatic mosaicism. Genes Dev.

[30] Dennis E S, Brettell R I (1990). DNA methylation of maize transposable elements is correlated with activity. Philos. Trans. R. Soc. Lond. B. Biol. Sci.

[31] Barlow D P (1993). Methylation and imprinting: from host defense to gene regulation?. Science.

[32] Yoder J A, Walsh C P, Bestor T H (1997). Cytosine methylation and the ecology of intragenomic parasites. Trends Genet.

[33] Bourc'his D, Bestor T H (2004). Meiotic catastrophe and retrotransposon reactivation in male germ cells lacking Dnmt3L. Nature.

[34] Obbard D J, Gordon K H, Buck A H, Jiggins F M (2009). The evolution of RNAi as a defence against viruses and transposable elements. Philos. Trans. R. Soc. Lond. B. Biol. Sci.

[35] Sijen T, Plasterk R H (2003). Transposon silencing in the Caenorhabditis elegans germ line by natural RNAi. Nature.

[36] Vagin V V, Klenov M S, Kalmykova A I, Stolyarenko A D, Kotelnikov R N, Gvozdev V A (2004). The RNA interference proteins and vasa locus are involved in the silencing of retrotransposons in the female germline of Drosophila melanogaster. RNA Biol.

[37] Engels W R (1996). P elements in Drosophila. Curr. Top. Microbiol. Immunol.

[38] Schumann G G (2007). APOBEC3 proteins: major players in intracellular defence against LINE-1-mediated retrotransposition. Biochem. Soc. Trans.

[39] Stetson D B, Ko J S, Heidmann T, Medzhitov R (2008). Trex1 prevents cell-intrinsic initiation of autoimmunity. Cell.

[40] Sinzelle L, Izsvak Z, Ivics Z (2009). Molecular domestication of transposable elements: from detrimental parasites to useful host genes. Cell. Mol. Life Sci.

[41] Sakano H, Huppi K, Heinrich G, Tonegawa S (1979). Sequences at the somatic recombination sites of immunoglobulin light-chain genes. Nature.

[42] van Gent D C, Mizuuchi K, Gellert M (1996). Similarities between initiation of V(D)J recombination and retroviral integration. Science.

[43] Kennedy A K, Guhathakurta A, Kleckner N, Haniford D B (1998). Tn10 transposition *via* a DNA hairpin intermediate. Cell.

[44] Agrawal A, Eastman Q M, Schatz D G (1998). Transposition mediated by RAG1 and RAG2 and its implications for the evolution of the immune system. Nature.

[45] Melek M, Gellert M, van Gent D C (1998). Rejoining of DNA by the RAG1 and RAG2 proteins. Science.

[46] Casola C, Hucks D, Feschotte C (2008). Convergent domestication of pogo-like transposases into centromere-binding proteins in fission yeast and mammals. Mol. Biol. Evol.

[47] Lee S H, Oshige M, Durant S T, Rasila K K, Williamson E A, Ramsey H, Kwan L, Nickoloff J A, Hromas R (2005). The SET domain protein Metnase mediates foreign DNA integration and links integration to nonhomologous end-joining repair. Proc. Natl. Acad. Sci. USA.

[48] Nekrutenko A, Li W H (2001). Transposable elements are found in a large number of human protein-coding genes. Trends Genet.

[49] Jordan I K, Rogozin I B, Glazko G V, Koonin E V (2003). Origin of a substantial fraction of human regulatory sequences from transposable elements. Trends Genet.

[50] Beauregard A, Curcio M J, Belfort M (2008). The take and give between retrotransposable elements and their hosts. Annu. Rev. Genet.

[51] Goodier J L, Kazazian H H (2008). Retrotransposons revisited: the restraint and rehabilitation of parasites. Cell.

[52] Ostertag E M, Madison B B, Kano H (2007). Mutagenesis in rodents using the L1 retrotransposon. Genome Biol.

[53] Feschotte C, Jiang N, Wessler S R (2002). Plant transposable elements: where genetics meets genomics. Nat. Rev. Genet.

[54] Bureau T E, Ronald P C, Wessler S R (1996). A computer-based systematic survey reveals the predominance of small inverted-repeat elements in wild-type rice genes. Proc. Natl. Acad. Sci. USA.

[55] Buisine N, Tang C M, Chalmers R (2002). Transposon-like Correia elements: structure, distribution and genetic exchange between pathogenic Neisseria sp. FEBS Lett.

[56] De Gregorio E, Silvestro G, Petrillo M, Carlomagno M S, Di Nocera P P (2005). Enterobacterial repetitive intergenic consensus sequence repeats in yersiniae: genomic organization and functional properties. J. Bacteriol.

[57] Robertson H M (1993). The mariner transposable element is widespread in insects. Nature.

[58] Plasterk R H, Izsvak Z, Ivics Z (1999). Resident aliens: the Tc1/mariner superfamily of transposable elements. Trends Genet.

[59] Arkhipova I R, Meselson M (2005). Diverse DNA transposons in rotifers of the class Bdelloidea. Proc. Natl. Acad. Sci. USA.

[60] Emmons S W, Yesner L, Ruan K S, Katzenberg D (1983). Evidence for a transposon in Caenorhabditis elegans. Cell.

[61] Collins J, Forbes E, Anderson P (1989). The Tc3 family of transposable genetic elements in Caenorhabditis elegans. Genetics.

[62] Franz G, Savakis C (1991). Minos, a new transposable element from Drosophila hydei, is a member of the Tc1-like family of transposons. Nucleic Acids Res.

[63] Hartl D (2001). Discovery of the transposable element mariner. Genetics.

[64] Medhora M, Maruyama K, Hartl D L (1991). Molecular and functional analysis of the mariner mutator element Mos1 in Drosophila. Genetics.

[65] Barry E G, Witherspoon D J, Lampe D J (2004). A bacterial genetic screen identifies functional coding sequences of the insect mariner transposable element Famar1 amplified from the genome of the earwig, Forficula auricularia. Genetics.

[66] Yang G, Weil C F, Wessler S R (2006). A rice Tc1/mariner-like element transposes in yeast. Plant Cell.

[67] Daboussi M J, Langin T, Brygoo Y (1992). Fot1, a new family of fungal transposable elements. Mol. Gen. Genet.

[68] Langin T, Capy P, Daboussi M J (1995). The transposable element impala, a fungal member of the Tc1-mariner superfamily. Mol. Gen. Genet.

[69] Feng X, Colloms S D (2007). *In vitro* transposition of ISY100, a bacterial insertion sequence belonging to the Tc1/mariner family. Mol. Microbiol.

[70] Munoz-Lopez M, Siddique A, Bischerour J, Lorite P, Chalmers R, Palomeque T (2008). Transposition of Mboumar-9: identification of a new naturally active mariner-family transposon. J. Mol. Biol.

[71] Radice A D, Bugaj B, Fitch D H, Emmons S W (1994). Widespread occurrence of the Tc1 transposon family: Tc1-like transposons from teleost fish. Mol. Gen. Genet.

[72] Lampe D J, Churchill M E, Robertson H M (1996). A purified mariner transposase is sufficient to mediate transposition *in vitro*. EMBO J.

[73] Miskey C, Izsvak Z, Plasterk R H, Ivics Z (2003). The Frog Prince: a reconstructed transposon from Rana pipiens with high transpositional activity in vertebrate cells. Nucleic Acids Res.

[74] Liu D, Bischerour J, Siddique A, Buisine N, Bigot Y, Chalmers R (2007). The human SETMAR protein preserves most of the activities of the ancestral Hsmar1 transposase. Mol. Cell. Biol.

[75] Miskey C, Papp B, Mates L, Sinzelle L, Keller H, Izsvak Z, Ivics Z (2007). The ancient mariner sails again: transposition of the human Hsmar1 element by a reconstructed transposase and activities of the SETMAR protein on transposon ends. Mol. Cell. Biol.

[76] Cordaux R, Udit S, Batzer M A, Feschotte C (2006). Birth of a chimeric primate gene by capture of the transposase gene from a mobile element. Proc. Natl. Acad. Sci. USA.

[77] Leroy H, Castagnone-Sereno P, Renault S, Auge-Gouillou C, Bigot Y, Abad P (2003). Characterization of Mcmar1, a mariner-like element with large inverted terminal repeats(ITRs) from the phytoparasitic nematode Meloidogyne chitwoodi. Gene.

[78] Brillet B, Bigot Y, Auge-Gouillou C (2007). Assembly of the Tc1 and mariner transposition initiation complexes depends on the origins of their transposase DNA binding domains. Genetica.

[79] Claudianos C, Brownlie J, Russell R, Oakeshott J, Whyard S (2002). maT--a clade of transposons intermediate between mariner and Tc1. Mol. Biol. Evol.

[80] Shao H, Tu Z (2001). Expanding the diversity of the IS630-Tc1-mariner superfamily: discovery of a unique DD37E transposon and reclassification of the DD37D and DD39D transposons. Genetics.

[81] Vos J C, van Luenen H G, Plasterk R H (1993). Characterization of the Caenorhabditis elegans Tc1 transposase *in vivo* and *in vitro*. Genes Dev.

[82] Doak T G, Doerder F P, Jahn C L, Herrick G (1994). A proposed superfamily of transposase genes: transposon-like elements in ciliated protozoa and a common "D35E" motif. Proc. Natl. Acad. Sci. USA.

[83] Ivics Z, Hackett P B, Plasterk R H, Izsvak Z (1997). Molecular reconstruction of Sleeping Beauty, a Tc1-like transposon from fish, and its transposition in human cells. Cell.

[84] Sinzelle L, Pollet N, Bigot Y, Mazabraud A (2005). Characterization of multiple lineages of Tc1-like elements within the genome of the amphibian Xenopus tropicalis. Gene.

[85] Coy M R, Tu Z (2005). Gambol and Tc1 are two distinct families of DD34E transposons: analysis of the Anopheles gambiae genome expands the diversity of the IS630-Tc1-mariner superfamily. Insect Mol. Biol.

[86] Jarvik T, Lark K G (1998). Characterization of Soymar1, a mariner element in soybean. Genetics.

[87] Morisato D, Kleckner N (1987). Tn10 transposition and circle formation *in vitro*. Cell.

[88] Bushman F D, Craigie R (1991). Activities of human immunodeficiency virus(HIV) integration protein *in vitro*: specific cleavage and integration of HIV DNA. Proc. Natl. Acad. Sci. USA.

[89] Vos J C, De Baere I, Plasterk R H (1996). Transposase is the only nematode protein required for *in vitro* transposition of Tc1. Genes Dev.

[90] Allingham J S, Haniford D B (2002). Mechanisms of metal ion action in Tn10 transposition. J. Mol. Biol.

[91] Tosi L R, Beverley S M (2000). cis and trans factors affecting Mos1 mariner evolution and transposition *in vitro*, and its potential for functional genomics. Nucleic Acids Res.

[92] Miskey C, Izsvak Z, Kawakami K, Ivics Z (2005). DNA transposons in vertebrate functional genomics. Cell. Mol. Life Sci.

[93] Haber J E (2000). Partners and pathwaysrepairing a double-strand break. Trends Genet.

[94] Plasterk R H (1991). The origin of footprints of the Tc1 transposon of Caenorhabditis elegans. EMBO J.

[95] van Luenen H G, Colloms S D, Plasterk R H (1994). The mechanism of transposition of Tc3 in C. elegans. Cell.

[96] Lohe A R, Hartl D L (1996). Autoregulation of mariner transposase activity by overproduction and dominant-negative complementation. Mol. Biol. Evol.

[97] Lampe D J, Grant T E, Robertson H M (1998). Factors affecting transposition of the Himar1 mariner transposon *in vitro*. Genetics.

[98] Lohe A R, Moriyama E N, Lidholm D A, Hartl D L (1995). Horizontal transmission, vertical inactivation, and stochastic loss of mariner-like transposable elements. Mol. Biol. Evol.

[99] Lohe A R, De Aguiar D, Hartl D L (1997). Mutations in the mariner transposase: the D,D(35)E consensus sequence is nonfunctional. Proc. Natl. Acad. Sci. USA.

[100] Hollister J D, Gaut B S (2009). Epigenetic silencing of transposable elements: A trade-off between reduced transposition and deleterious effects on neighboring gene expression. Genome Res.

[101] Vastenhouw N L, Plasterk R H (2004). RNAi protects the Caenorhabditis elegans germline against transposition. Trends Genet.

[102] Slotkin R K, Martienssen R (2007). Transposable elements and the epigenetic regulation of the genome. Nat. Rev. Genet.

[103] Hartl D L, Lohe A R, Lozovskaya E R (1997). Modern thoughts on an ancyent marinere: function, evolution, regulation. Annu. Rev. Genet.

[104] Casse N, Bui Q T, Nicolas V, Renault S, Bigot Y, Laulier M (2006). Species sympatry and horizontal transfers of Mariner transposons in marine crustacean genomes. Mol. Phylogenet. Evol.

[105] Lampe D J, Witherspoon D J, Soto-Adames F N, Robertson H M (2003). Recent horizontal transfer of mellifera subfamily mariner transposons into insect lineages representing four different orders shows that selection acts only during horizontal transfer. Mol. Biol. Evol.

[106] Robertson H M, Lampe D J (1995). Recent horizontal transfer of a mariner transposable element among and between Diptera and Neuroptera. Mol. Biol. Evol.

[107] Laha T, Loukas A, Wattanasatitarpa S, Somprakhon J, Kewgrai N, Sithithaworn P, Kaewkes S, Mitreva M, Brindley P J (2007). The bandit, a New DNA Transposon from a Hookworm-Possible Horizontal Genetic Transfer between Host and Parasite. PLoS. Negl. Trop. Dis.

[108] Houck M A, Clark J B, Peterson K R, Kidwell M G (1991). Possible horizontal transfer of Drosophila genes by the mite Proctolaelaps regalis. Science.

[109] Mates L, Izsvak Z, Ivics Z (2007). Technology transfer from worms and flies to vertebrates: transposition-based genome manipulations and their future perspectives. Genome Biol.

[110] Mates L, Chuah M K, Belay E, Jerchow B, Manoj N, Acosta-Sanchez A, Grzela D P, Schmitt A, Becker K, Matrai J, Ma L, Samara-Kuko E, Gysemans C, Pryputniewicz D, Miskey C, Fletcher B, Vandendriessche T, Ivics Z, Izsvak Z (2009). Molecular evolution of a novel hyperactive Sleeping Beauty transposase enables robust stable gene transfer in vertebrates. Nat. Genet.

[111] Huang X, Wilber A C, Bao L, Tuong D, Tolar J, Orchard P J, Levine B L, June C H, McIvor R S, Blazar B R, Zhou X (2006). Stable gene transfer and expression in human primary T cells by the Sleeping Beauty transposon system. Blood.

[112] Grabher C, Henrich T, Sasado T, Arenz A, Wittbrodt J, Furutani-Seiki M (2003). Transposon-mediated enhancer trapping in medaka. Gene.

[113] Sinzelle L, Vallin J, Coen L, Chesneau A, Du Pasquier D, Pollet N, Demeneix B, Mazabraud A (2006). Generation of trangenic Xenopus laevis using the Sleeping Beauty transposon system. Transgenic Res.

[114] Kitada K, Ishishita S, Tosaka K, Takahashi R, Ueda M, Keng V W, Horie K, Takeda J (2007). Transposon-tagged mutagenesis in the rat. Nat. Methods.

[115] Dupuy A J, Akagi K, Largaespada D A, Copeland N G, Jenkins N A (2005). Mammalian mutagenesis using a highly mobile somatic Sleeping Beauty transposon system. Nature.

[116] Collier L S, Carlson C M, Ravimohan S, Dupuy A J, Largaespada D A (2005). Cancer gene discovery in solid tumours using transposon-based somatic mutagenesis in the mouse. Nature.

[117] Dupuy A J, Jenkins N A, Copeland N G (2006). Sleeping beauty: a novel cancer gene discovery tool. Hum. Mol. Genet.

[118] Zagoraiou L, Drabek D, Alexaki S, Guy J A, Klinakis A G, Langeveld A, Skavdis G, Mamalaki C, Grosveld F, Savakis C (2001). *In vivo* transposition of Minos, a Drosophila mobile element, in mammalian tissues. Proc. Natl. Acad. Sci. USA.

[119] Drabek D, Zagoraiou L, deWit T, Langeveld A, Roumpaki C, Mamalaki C, Savakis C, Grosveld F (2003). Transposition of the Drosophila hydei Minos transposon in the mouse germ line. Genomics.

[120] Sasakura Y, Awazu S, Chiba S, Satoh N (2003). Germ-line transgenesis of the Tc1/mariner superfamily transposon Minos in Ciona intestinalis. Proc. Natl. Acad. Sci. USA.

[121] Maier T M, Pechous R, Casey M, Zahrt T C, Frank D W (2006). *In vivo* Himar1-based transposon mutagenesis of Francisella tularensis. Appl. Environ. Microbiol.

[122] Liu Z M, Tucker A M, Driskell L O, Wood D O (2007). Mariner-based transposon mutagenesis of Rickettsia prowazekii. Appl. Environ. Microbiol.

[123] Wu Q, Pei J, Turse C, Ficht T A (2006). Mariner mutagenesis of Brucella melitensis reveals genes with previously uncharacterized roles in virulence and survival. BMC Microbiol.

[124] Keravala A, Liu D, Lechman E R, Wolfe D, Nash J A, Lampe D J, Robbins P D (2006). Hyperactive Himar1 transposase mediates transposition in cell culture and enhances gene expression *in vivo*. Hum. Gene Ther.

[125] Sarkar A, Sim C, Hong Y S, Hogan J R, Fraser M J, Robertson H M, Collins F H (2003). Molecular evolutionary analysis of the widespread piggyBac transposon family and related "domesticated" sequences. Mol. Genet. Genomics.

[126] Cary L C, Goebel M, Corsaro B G, Wang H G, Rosen E, Fraser M J (1989). Transposon mutagenesis of baculoviruses: analysis of Trichoplusia ni transposon IFP2 insertions within the FP-locus of nuclear polyhedrosis viruses. Virology.

[127] Cadinanos J, Bradley A (2007). Generation of an inducible and optimized piggyBac transposon system. Nucleic Acids Res.

[128] Balu B, Shoue D A, Fraser M J, Adams J H (2005). High-efficiency transformation of Plasmodium falciparum by the lepidopteran transposable element piggyBac. Proc. Natl. Acad. Sci. USA.

[129] Gonzalez-Estevez C, Momose T, Gehring W J, Salo E (2003). Transgenic planarian lines obtained by electroporation using transposon-derived vectors and an eye-specific GFP marker. Proc. Natl. Acad. Sci. USA.

[130] Lukacsovich T, Hamada N, Miyazaki S, Kimpara A, Yamamoto D (2008). A new versatile gene-trap vector for insect transgenics. Arch. Insect. Biochem. Physiol.

[131] Yusa K, Rad R, Takeda J, Bradley A (2009). Generation of transgene-free induced pluripotent mouse stem cells by the piggyBac transposon. Nat. Methods.

[132] Wilson M H, Coates C J, George A L (2007). PiggyBac transposon-mediated gene transfer in human cells. Mol. Ther.

[133] Alphey L (2002). Re-engineering the sterile insect technique. Insect Biochem. Mol. Biol.

[134] Robinson A S, Franz G, Atkinson P W (2004). Insect transgenesis and its potential role in agriculture and human health. Insect Biochem. Mol. Biol.

[135] Marec F, Neven L G, Robinson A S, Vreysen M, Goldsmith M R, Nagaraju J, Franz G (2005). Development of genetic sexing strains in Lepidoptera: from traditional to transgenic approaches. J. Econ. Entomol.

[136] Kempken F, Windhofer F (2001). The hAT family: a versatile transposon group common to plants, fungi, animals, and man. Chromosoma.

[137] Aravind L (2000). The BED finger, a novel DNA-binding domain in chromatin-boundary-element-binding proteins and transposases. Trends Biochem. Sci.

[138] Kawakami K, Shima A (1999). Identification of the Tol2 transposase of the medaka fish Oryzias latipes that catalyzes excision of a nonautonomous Tol2 element in zebrafish Danio rerio. Gene.

[139] Kawakami K, Shima A, Kawakami N (2000). Identification of a functional transposase of the Tol2 element, an Ac-like element from the Japanese medaka fish, and its transposition in the zebrafish germ lineage. Proc. Natl. Acad. Sci. USA.

[140] Koga A (2004). Transposition mechanisms and biotechnology applications of the medaka fish Tol2 transposable element. Adv. Biophys.

[141] Kawakami K, Noda T (2004). Transposition of the Tol2 element, an Ac-like element from the Japanese medaka fish Oryzias latipes, in mouse embryonic stem cells. Genetics.

[142] Choo B G, Kondrichin I, Parinov S, Emelyanov A, Go W, Toh W C, Korzh V (2006). Zebrafish transgenic Enhancer TRAP line database(ZETRAP). BMC Dev. Biol.

[143] Kawakami K (2007). Tol2: a versatile gene transfer vector in vertebrates. Genome Biol.

[144] Balciunas D, Wangensteen K J, Wilber A, Bell J, Geurts A, Sivasubbu S, Wang X, Hackett P B, Largaespada D A, McIvor R S, Ekker S C (2006). Harnessing a high cargo-capacity transposon for genetic applications in vertebrates. PLoS. Genet.

[145] Urasaki A, Morvan G, Kawakami K (2006). Functional dissection of the Tol2 transposable element identified the minimal cis-sequence and a highly repetitive sequence in the subterminal region essential for transposition. Genetics.

[146] Kotani T, Nagayoshi S, Urasaki A, Kawakami K (2006). Transposon-mediated gene trapping in zebrafish. Methods.

[147] Kawakami K, Takeda H, Kawakami N, Kobayashi M, Matsuda N, Mishina M (2004). A transposon-mediated gene trap approach identifies developmentally regulated genes in zebrafish. Dev. Cell.

[148] Hamlet M R, Yergeau D A, Kuliyev E, Takeda M, Taira M, Kawakami K, Mead P E (2006). Tol2 transposon-mediated transgenesis in Xenopus tropicalis. Genesis.

[149] Sato Y, Kasai T, Nakagawa S, Tanabe K, Watanabe T, Kawakami K, Takahashi Y (2007). Stable integration and conditional expression of electroporated transgenes in chicken embryos. Dev. Biol.

[150] Thomas C E, Ehrhardt A, Kay M A (2003). Progress and problems with the use of viral vectors for gene therapy. Nat. Rev. Genet.

[151] Manno C S, Pierce G F, Arruda V R, Glader B, Ragni M, Rasko J J, Ozelo M C, Hoots K, Blatt P, Konkle B, Dake M, Kaye R, Razavi M, Zajko A, Zehnder J, Rustagi P K, Nakai H, Chew A, Leonard D, Wright J F, Lessard R R, Sommer J M, Tigges M, Sabatino D, Luk A, Jiang H, Mingozzi F, Couto L, Ertl H C, High K A, Kay M A (2006). Successful transduction of liver in hemophilia by AAV-Factor IX and limitations imposed by the host immune response. Nat. Med.

[152] Grimm D, Kern A, Rittner K, Kleinschmidt J A (1998). Novel tools for production and purification of recombinant adenoassociated virus vectors. Hum. Gene Ther.

[153] Tiscornia G, Singer O, Verma I M (2006). Production and purification of lentiviral vectors. Nat. Protoc.

[154] Wu X, Li Y, Crise B, Burgess S M (2003). Transcription start regions in the human genome are favored targets for MLV integration. Science.

[155] Wu X, Luke B T, Burgess S M (2006). Redefining the common insertion site. Virology.

[156] Ivics Z, Li M A, Mates L, Boeke J D, Nagy A, Bradley A, Izsvak Z (2009). Transposon-mediated genome manipulation in vertebrates. Nat. Methods.

[157] Aronovich E L, Bell J B, Belur L R, Gunther R, Koniar B, Erickson D C, Schachern P A, Matise I, McIvor R S, Whitley C B, Hackett P B (2007). Prolonged expression of a lysosomal enzyme in mouse liver after Sleeping Beauty transposon-mediated gene delivery: implications for non-viral gene therapy of mucopolysaccharidoses. J. Gene. Med.

[158] Ohlfest J R, Frandsen J L, Fritz S, Lobitz P D, Perkinson S G, Clark K J, Nelsestuen G, Key N S, McIvor R S, Hackett P B, Largaespada D A (2005). Phenotypic correction and long-term expression of factor VIII in hemophilic mice by immunotolerization and nonviral gene transfer using the Sleeping Beauty transposon system. Blood.

[159] Yant S R, Wu X, Huang Y, Garrison B, Burgess S M, Kay M A (2005). High-resolution genome-wide mapping of transposon integration in mammals. Mol. Cell. Biol.

[160] Bushman F, Lewinski M, Ciuffi A, Barr S, Leipzig J, Hannenhalli S, Hoffmann C (2005). Genome-wide analysis of retroviral DNA integration. Nat. Rev. Microbiol.

[161] Izsvak Z, Ivics Z, Plasterk R H (2000). Sleeping Beauty, a wide host-range transposon vector for genetic transformation in vertebrates. J. Mol. Biol.

[162] Ding S, Wu X, Li G, Han M, Zhuang Y, Xu T (2005). Efficient transposition of the piggyBac(PB) transposon in mammalian cells and mice. Cell.

[163] Zayed H, Izsvak Z, Walisko O, Ivics Z (2004). Development of hyperactive sleeping beauty transposon vectors by mutational analysis. Mol. Ther.

[164] Vigdal T J, Kaufman C D, Izsvak Z, Voytas D F, Ivics Z (2002). Common physical properties of DNA affecting target site selection of sleeping beauty and other Tc1/mariner transposable elements. J. Mol. Biol.

[165] Liu G, Geurts A M, Yae K, Srinivasan A R, Fahrenkrug S C, Largaespada D A, Takeda J, Horie K, Olson W K, Hackett P B (2005). Target-site preferences of Sleeping Beauty transposons. J. Mol. Biol.

[166] Li X, Harrell R A, Handler A M, Beam T, Hennessy K, Fraser M J (2005). piggyBac internal sequences are necessary for efficient transformation of target genomes. Insect. Mol. Biol.

[167] Geurts A M, Hackett C S, Bell J B, Bergemann T L, Collier L S, Carlson C M, Largaespada D A, Hackett P B (2006). Structure-based prediction of insertion-site preferences of transposons into chromosomes. Nucleic Acids Res.

[168] Wallrath L L, Elgin S C (1995). Position effect variegation in Drosophila is associated with an altered chromatin structure. Genes Dev.

[169] Bancroft I, Dean C (1993). Transposition pattern of the maize element Ds in Arabidopsis thaliana. Genetics.

[170] Dooner H K, Keller J, Harper E, Ralston E (1991). Variable Patterns of Transposition of the Maize Element Activator in Tobacco. Plant Cell.

[171] Belzile F, Yoder J I (1992). Pattern of somatic transposition in a high copy Ac tomato line. Plant J.

[172] Knapp S, Larondelle Y, Rossberg M, Furtek D, Theres K (1994). Transgenic tomato lines containing Ds elements at defined genomic positions as tools for targeted transposon tagging. Mol. Gen. Genet.

[173] Wu S C, Meir Y J, Coates C J, Handler A M, Pelczar P, Moisyadi S, Kaminski J M (2006). piggyBac is a flexible and highly active transposon as compared to sleeping beauty, Tol2, and Mos1 in mammalian cells. Proc. Natl. Acad. Sci. USA.

